# Physical activity, sedentary behaviour and health-related quality of life in 929 women with primary Raynaud’s phenomenon

**DOI:** 10.1007/s10067-025-07416-w

**Published:** 2025-04-17

**Authors:** Saskia Corine van de Zande, Karin Eijkelenkamp, Amaal Eman Abdulle, Andries Jan Smit, Johannes Zwerver, Inge van den Akker-Scheek, Douwe Johannes Mulder

**Affiliations:** 1 Division of Vascular Medicine, Department of Internal Medicine, University Medical Center Groningen, University of Groningen, Groningen, The Netherlands; 2 Department of Endocrinology and Metabolic Diseases, University Medical Center Groningen, University of Groningen, Groningen, The Netherlands; 3 Department of Orthopaedics, University Medical Center Groningen, University of Groningen, Groningen, The Netherlands; 4 Center for Human Movement Sciences, University Medical Center Groningen, University of Groningen, Groningen, The Netherlands; 5 Sports Valley, Gelderse Vallei Hospital, Ede, The Netherlands

**Keywords:** Health-related quality of life, Physical activity, Raynaud's phenomenon

## Abstract

**Introduction:**

Women with primary Raynaud’s phenomenon (RP) often experience a decreased health-related quality of life (HRQoL). A physically active lifestyle could improve vascular function and HRQoL.

**Objective:**

This study aimed to quantify the amount and type of physical activity (PA) and sedentary behaviour, as well as HRQoL and stress, in women with primary RP from a large population-based cohort (Lifelines).

**Methods:**

A total of 19,820 adult (≥ 18 years) women from the Lifelines cohort were included; 929 of these were classified as having RP based on the connective tissue disease (CTD) screening questionnaire. Participant characteristics, data on PA and sedentary behaviour, HRQoL and stress were retrieved from the database.

**Results:**

Women with RP reported 300 min/week minutes of moderate-to-vigorous physical activity (MVPA), which was more than women without RP (255 min/week, *p* ≤ .001). Women (74%) with RP complied to health enhancing PA guidelines (70% of women without RP, *p* = .003). Sedentary time was comparable. Women with RP had a low score on almost all eight domains of the HRQoL questionnaire. The Long-term Difficulties Inventory (LDI) showed a high stress level in the RP group (*p* < .001).

**Conclusion:**

Most women with RP reported to spent a sufficient amount of time on MVPA and thus comply to health enhancing PA guidelines. The PA and sedentary behaviour of women with RP seems comparable to that of women without RP. However, HRQoL was lower and stress levels were higher in women with RP; more research is needed to elucidate the relation between PA and HRQoL in RP.

**Table:** 

**Key Points**
• *Experiencing symptoms of Raynaud’s phenomenon seem no obstacle for being physically active.* • *Focus on women with symptoms of Raynaud’s phenomenon in a large cohort.* • *Nearly 75% of the women with symptoms of Raynaud’s phenomenon comply to the physical activity guidelines.* • *Women with symptoms of Raynaud’s phenomenon have low levels of health-related quality of life and experience a high stress level.*

**Supplementary Information:**

The online version contains supplementary material available at 10.1007/s10067-025-07416-w.

## Introduction

Raynaud’s phenomenon (RP) is a common vasospastic disorder affecting extremities including digits, toes, nose and earlobes in response to cold or emotional stress [1, 2]. RP occurs especially in young females, mostly primary (idiopathic) and benign; however, RP also occurs secondary to an underlying autoimmune disease. Although it is reported that mild primary RP rarely interferes with daily activities, it is overlooked that the overall effect on health-related quality of life (HRQoL) is reduced due to experienced pain, anxiety and/or depression [3–6]. The goal of treatment of primary RP is to reduce symptoms and improve HRQoL. Current treatment consists of well-known non-pharmacological measures, such as patient education, maintaining body warmth and avoiding other triggers for RP attacks [7]. However, the effect of promoting physical activity (PA) is largely unknown. Research has mostly been performed on patients that sought medical attention for their RP symptoms. However, little is known about HRQoL and PA of women who did not seek medical attention. It is well established that PA may have a positive impact on quality of life (QoL) and well-being in healthy adults [8]. PA was shown to have an additional positive effect on vascular function, especially on endothelial function [9, 10]. Structural PA may also improve arterial remodelling in humans [10], making it a potential relevant intervention for people suffering from the restrains of RP in daily life.

PA guidelines issued by the World Health Organization (WHO) recommend physical activity of at least 150–300 min of moderate-intensity or 75–150 min of vigorous-intensity, or an equivalent combination, per week. With the addition of muscle strengthening activities involving all major muscle groups, at least twice a week and limit time spent sedentary [11]. As it is known that in the general population compliance to health-enhancing PA guidelines is low, promotion of PA could be an important non-pharmacological intervention to reduce symptoms of RP, improve HRQoL and consequently to prevent the need for medical attention and unnecessary pharmacologic treatment. Therefore, the aim of this study was to quantify the amount of PA and sedentary behaviour, as well as HRQoL and stress, in women from a large population-based cohort (Lifelines) with symptoms of primary RP, and to compare this to the behaviour of women without RP symptoms.

## Materials and methods

Lifelines is a multi-disciplinary, prospective, population-based cohort study examining, in a unique three-generation design, health and health-related behaviours of 167,729 persons living in the north of The Netherlands [12]. It employs a broad range of investigative procedures in assessing biomedical, socio-demographic, behavioural, physical and psychological factors that contribute to health and disease of the general population, with a special focus on multi-morbidity and complex genetics [13]. All Lifelines participants provided written informed consent before participating in the study. The Lifelines protocol was approved by the UMCG Medical Ethical Committee under number 2007/152 (www.lifelines.nl). At baseline visit for Lifelines, all questionnaires were conducted and measurements were performed by research nurses. Height (in cm), mass (in kg), body mass index (BMI) (in kg/m^2^), waist and hip circumference (in cm) and smoking were defined by amount of pack years.

### Participants

For the current study, we examined prospectively collected data of the Lifelines baseline visit from women ≥ 18 years old who completed the connective tissue disease (CTD) screening questionnaire and who reported no comorbidities. This is a validated 30-item questionnaire to detect potential CTDs, including questions on RP. Based on two questions of the CTD screening questionnaire, the population was divided into those with RP and those without RP, as described before [2]. PA, HrQoL and stress were assessed by validated questionnaires. The SQUASH for activity of an average week in the past month [14], in which the outcome is total hours of PA per week as well as total hours of light, moderate and vigorous activities per week and a combination of the latter two combined into moderate-to-vigorous physical activity (MVPA). The Marshall sitting questionnaire determines sedentary time (minutes per day) for a weekday [14]. The Health Survey short form (SF-36) for HrQoL [15], the List of Threatening Experiences (LTE) [16] and the Long-term Difficulties Inventory (LDI) [17] determine occurrence of stressful life events and amount of chronic stress in the past 12 months, respectively. More detail of all questionnaires can be found in the supplementary file.

### Statistical analysis

For statistical analysis, we used IBM SPSS (version 23.0; IBM, Armonk, NY, USA). Categorical data is presented as number (percentage), normally distributed data is presented as mean (standard deviation (SD)) and median (interquartile range (IQR)) was used when data was not normally distributed. An independent samples *t*-test or, when normality was violated, a Mann–Whitney *U* test was used to determine whether there were any statistically significant differences in determined variables between groups (with and without RP). Univariate logistic regression analysis was performed to assess association between RP and PA, HRQoL and stress. For each statistically significant variable in the univariate analysis, a separate multivariate logistic regression (enter method) was performed to correct for potential confounders (i.e. age and BMI). *p*-values of < 0.05 were considered to be statistically significant.

## Results

The total population consisted of 19,820 women, 929 with RP and 18,891 without RP. Table 1 provides the characteristics of the study population. Women with RP were significantly younger compared to women without RP (*p* < 0.001), had a lower BMI (*p* < 0.001), a lower waist-hip ratio (*p* < 0.001), had a lower amount of packyears (*p* < 0.001) and a healthier diet (*p* = 0.043).

**Table 1:** Characteristics of participants with and without Raynaud’s phenomenon

	Raynaud’s phenomenon		No Raynaud’s phenomenon		
	*N*		*N*		*p*
Age (years; mean (SD))	929	39 (12)	18,891	42 (12)	< .001
BMI (kg/m^2^; mean (SD))	929	23.11 (3.39)	18,886	25.01 (4.10)	< .001
Waist circumference (cm; mean (SD))	929	79.6 (9.8)	18,887	84.4 (11.0)	< .001
Hip circumference (cm; mean (SD))	929	94.6 (9.1)	18,885	98.5 (9.9)	< .001
Waist-hip ratio; mean (SD)	929	0.84 (0.07)	18,885	0.86 (0.07)	< .001
Packyears (cigarettes only; median (IQR))	910	0 (0 – 3.0)	18,383	0 (0 – 4.75)	< .001
Current smoker (%yes)	136 (14.8%)		3199 (17.2%)		.060
Previous smoker (%yes)	265 (65.9%)		5972 (64.0%)		.429

*SD* standard deviation, *BMI* body mass index, *IQR* interquartile range

### Physical activity and sedentary behaviour

**Fig. 1:**
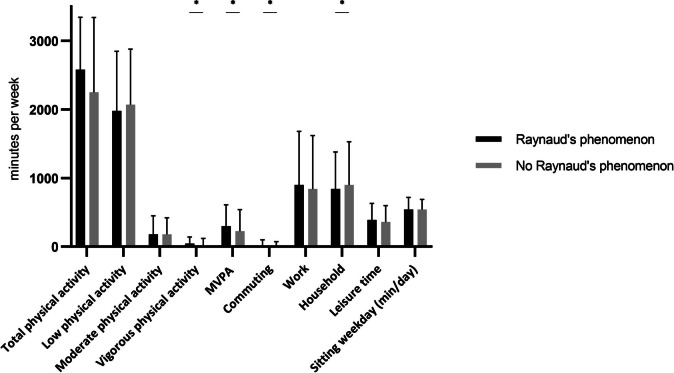
Physical activity and sedentary time of participants with and without Raynaud’s phenomenon

Figure 1 shows the results of PA and sedentary time of participants with and without RP. Women with RP had a total of 2580 min of PA per week; total minutes of MVPA were 300 min/week. For women without RP, the total minutes of PA were 2550 min per week and 255 MVPA minutes per week. There was a significant difference between women with and without RP (*p* < 0.001), mainly caused by a difference in vigorous activities (women with RP 45 min/week and women without RP 0 min/week, *p* < 0.001). The amount of moderate activities differed not significantly between both groups (women with RP 180 min/week and women without RP 180 min/week, *p* = 0.204). For detailed information, see table S1. Univariate and multivariate logistic regression (Tables 2 and 3) model showed that with correction for age and BMI as potential confounders, MVPA (in minutes per week) still showed a significant association with RP (*B* = 0.000133, OR = 1.000133 [1.000013–1.000254], *p* = 0.030).

**Table 2:** Univariate logistic regression analyses investigating the association between RP (yes/no) and the amount of PA, HRQoL and stress

	Univariate analysis		
	*B*	OR [95% CI]	*p*
Age	− .026	.975 [.969–.980]	< .001
BMI	− .153	.858 [.840–.877]	< .001
Current smoking	− .178	.837 [.695–1.01]	.060
MVPA	.000138	1.00038 [1.00002–1.000257]	.022
Total min/week	− .000021	.999979 [.999927–1.000031]	.422
LTE	.036	1.037 [.980–1.097]	.205
LDI	.113	1.120 [1.092–1.149]	< .001

*RP* Raynaud’s phenomenon, *PA* physical activity, *HRQoL* health related quality of life, *OR* odds ratio, *BMI* body mass index, *MVPA* moderate-to-vigorous physical activity, *LTE* list of threatening experiences, *LDI* Long-term Difficulties Inventory

**Table 3:** Multivariate logistic regression analysis investigating the association between RP (yes/no) and amount of MVPA

	Multivariate analysis		
	*B*	OR [95% CI]	*p*
MVPA (min/week)	.000133	1.000133 [1.000013–1.000254]	.030
Age	− .018	.982 [.976–.988]	< .001
BMI	− .146	.864 [.845–.884]	< .001

*RP* Raynaud’s phenomenon, *MVPA* moderate-to-vigorous physical activity, *OR* odds ratio, *BMI* body mass index correction for age and BMI as potential confounders

When we examined the activity in each of the four domains as assessed with the SQUASH questionnaire, women with RP spend 840 min/week in household activities. This was significantly less than women without RP (900 min/week, *p* < 0.001). On the other hand, women with RP spend 15 min/week in commuting while women without RP spend 5 min/week in commuting (*p* < 0.001).

Adherence to the guidelines for PA (150 min per week of MVPA) was 74% in the group of women with RP and 70% in the group of women without RP (*p* = 0.003).

Women with and without RP spend both 540 min/week sedentary. See Table S1 for all results of the SQUASH questionnaire and Marshall sitting questionnaire.

### Health-related quality of life and stress

Women with RP had a low score on almost all eight domains of the SF-36 questionnaire, and these were lower compared to women without RP (*p* < 0.05 for seven domains, domain physical functioning *p* = 0.181). Regarding stress levels (Table 4), the Long-term Difficulties Inventory (LDI) showed a high stress level in the RP group; median for both groups was 2; however, the RP group showed a more broad IQR (1–4 for the RP group versus 1–3 for the non RP group, *p* < 0.001). The multivariate logistic regression (Table 5) model showed that with correction for age and BMI as potential confounders, LDI still showed a significant association with RP yes/no (B = 0.096 OR = 1.101 [1.0–1.131], *p* ≤ 0.001).

**Table 4:** HRQoL and stress level of participants with and without RP

	Raynaud’s phenomenon (*N* = 919–921)	No Raynaud’s phenomenon (*N* = 18,680–18,751)	
	Median (IQR)	Median (IQR)	*p*
Physical functioning	100 (90–100)	100 (90–100)	.181
Role limitations physical health	100 (100–100)	100 (100–100)	< .001
Role limitations emotional problems	100 (100–100)	100 (100–100)	.012
Fatigue	77 (55–80)	75 (60–80)	< .001
Emotional wellbeing	80 (72–88)	84 (76–92)	< .001
Social functioning	100 (75–100)	100 (87.5–100)	< .001
Pain	90 (67.5–100)	100 (77.5–100)	< .001
General health	75 (65–85)	80 (70–90)	< .001
LTE	1 (0–2)	1 (0–1)	.226
LDI	2 (1–4)	2 (1–3)	< .001

*HRQoL* health-related quality of life, *RP* Raynaud’s phenomenon, *IQR* interquartile range, *LTE* list of threatening experiences, *LDI* Long-term Difficulties Inventory

**Table 5:** Multivariate logistic regression analysis investigating the association between RP (yes/no) and LDI score

	Multivariate analysis		
	*B*	OR [95% CI]	*p*
LDI	.096	1.101 [1.072–1.131]	< .001
Age	− .015	.985 [.979–.991]	< .001
BMI	− .141	.869 [.850–.888]	< .001

*RP* Raynaud’s phenomenon, *OR* Odds Ratio, *LDI* Long Term Difficulties Inventory, *BMI* Body Mass Index correction for age and BMI as potential confounders

## Discussion

This study quantified the amount of PA in a large population-based group of women with symptoms of primary RP. Although differences on a population level were small, we found that nearly 75% of these women met the PA guidelines (> 150 min MVPA) contrary to 70% of those without RP symptoms. This difference was driven by more vigorous activities in the RP group. Furthermore, women with symptoms of RP showed low HRQoL and high stress levels.

Based on earlier studies, we expected that women with RP would have a lower amount of PA compared to women without RP. These studies showed that RP attacks interfere with daily activities in patients with early systemic sclerosis (SSc) and that RP is a common barrier to PA [18, 19]. However, according to the results of the current study, more women in the RP group met the guidelines compared to women without RP. A possible explanation is that RP symptoms of the women in the Lifelines population are less severe compared to RP symptoms in patients seeking medical attention. Lower body mass is linked to an increased risk of RP [2], which could explain why more active individuals have a lower body mass. The current study found that BMI and waist-hip ratio were significantly lower in the RP group. However, the association of MVPA with RP remained similar when corrected for BMI, indicating that body composition is not the sole factor at play.

The SQUASH questionnaire lacks details about type of training: strength or endurance training. As RP is mostly manifest in the fingers and hands, research is needed to determine if local vascular function improves as well as central vascular function with PA or that more specific training of the hands is needed. It might be beneficial to perform more strength enhancing exercises for the forearms and hands in order to improve the vascular function. Green et al. [10] described that exercise training improves vascular function. Next to more strength enhancing exercise, reduction of sitting time could potentially alleviate symptoms. A 16-week intervention in people with an increased risk of cardiovascular disease showed that a reduction of sedentary behaviour leads to a significant increase of the resting diameter of the superficial femoral artery [20]. This might be beneficial in women with RP because the range for dilation and constriction becomes larger. Additionally, lifestyle advice should focus on decreasing sitting time through simple modifications in daily life putatively assisted by wearable activity trackers.

Our finding that women with RP show lower HRQoL and higher stress levels is in line with previous research [3, 5]. It is well acknowledged that emotional stress is an important etiological factor in developing RP [21]. Furthermore, another study showed that people make at least one adjustment to their life due to RP, and they report a significant improvement of HRQoL when people were asked to imagine a life without RP [5]. A systematic review of PA and quality of life showed that PA improves QoL for healthy adults (18–65 years) [8]. More insight in PA behaviour and HRQoL can be achieved by considering severity of RP symptoms.

A first limitation of this study is that self-reported questionnaires were used. The ‘diagnosis’ RP was based on the CTD questionnaire, and as such on self-reported complaints and not on examination by a medical professional. Also, severity of RP was not taken into account. However, the CTD questionnaire is a valid questionnaire to detect potential CTD’s. Secondly, the Lifelines cohort study does not use device-based measurements for the assessment of PA behaviour, which give more detailed information about the intensity and amount of PA behaviour [22]. However, the SQUASH questionnaire is a reliable and valid instrument to measure PA in a large population [23]. Finally, our study was purely observational. Therefore, as cross-sectional associations are reported here, this does not necessarily indicate causality.

In conclusion, having RP seems no obstacle for being physically active, although 25% of the women with RP symptoms do not meet the health-enhancing PA guidelines. More research is needed to elucidate the relation between PA and HRQoL in this group, preferably using objective measurement instruments to assess PA.

## Supplementary Information

Below is the link to the electronic supplementary material.

Supplementary file1 (DOCX 23.2 KB)

## Data Availability

The data underlying this article will be shared on reasonable request to the corresponding author.
