# Supplementary material for: Physical activity, sedentary behaviour and health-related quality of life in 929 women with primary Raynaud’s phenomenon
Source: Clin Rheumatol. 2025 Apr 17;44(6):2101–7. doi: 10.1007/s10067-025-07416-w (PMC12141168; doi:10.1007/s10067-025-07416-w)
Supplement: Supplementary file 1 — Supplementary file1 (DOCX 23.2 KB) [file 10067_2025_7416_MOESM1_ESM.docx]

**Supplementary Information**

*Participants*

The connective tissue disease (CTD) screening questionnaire was filled out at baseline. This is a validated 30-item questionnaire to detect potential CTD’s, which also included questions on RP. Based on the first two questions of the CTD screening questionnaire the population was divided into those with RP and those without RP. The following applied: question 1: ‘Are your fingers unusually sensitive to the cold?’, question 2: ‘Have your fingers ever shown any unusual colour changes in the cold?’, if yes: question 2a: ‘If your fingers have ever shown any unusual colour changes in the cold, was the colour white?’, question 2b: ‘If your fingers have ever shown any unusual colour changes in the cold, was the colour blue?’ and question 2c: ‘If your fingers have ever shown any unusual colour changes in the cold, was the colour red?’. Participants were classified as having RP when they answered question one and two with ‘yes’ and reported biphasic or triphasic discoloration of the fingers i.e. a ‘yes’ on question 2b or 2c.

*Physical activity*

The SQUASH questionnaire (Dutch version) asks participants to estimate the activity of an average week in the past month. It is a reliable and valid questionnaire to measure the PA level in an adult population [1]. The questionnaire consists of four different domains: commuting activity, work activity, household activity, and leisure time activity (walking, biking, gardening, doing odd jobs, and sports). Participants were asked to fill out the type and amount of activity in each domain. Each activity has a certain metabolic equivalence of task (MET) value, based on Ainsworth’s compendium of physical activity [2] . With the age of the participant taken into account, activities were divided into light, moderate and vigorous activities. Outcome of the SQUASH questionnaire is the total hours of PA per week as well as the total hours of light, MVPA per week and a combination of the latter two. Additionally, the amount of activity in each of the four domains can be assessed. Moreover, it allows to determine whether a participant complies with the minimal recommended amount of MVPA of 150 min/week [3].

*Sedentary behaviour*

The Marshall sitting questionnaire determines the sedentary time (minutes per day) for a weekday [4]. The questionnaire has five items of sitting time spent per day: traveling, work, watching television, using a computer and during leisure time. Participants were asked to fill out the amount of hours and minutes per day they spent in the different domains for a weekday and for a day in the weekend. Total sitting time per day is calculated by adding all the hours and minutes of sitting in the different domains. In this study, a weekday was used because, according to Marshall [4], measurement properties are acceptable for a weekday because activities are likely to be more routine compared to a day in the weekend.

*Health-related quality of life and stress*

HRQoL was assessed by using the Health Survey short form (SF-36) [5]. It consists of 36 items categorized into 8 different domains (emotional well-being, energy/fatigue, general health, role limitations due to emotional problems, role limitations due to physical health, physical functioning, social functioning). Outcome is on a 100-point scale in which a higher score indicates a better HRQoL.

The List of Threatening Experiences (LTE) [6] and the Long-term Difficulties Inventory (LDI) [7] determines the occurrence of stressful life events and the amount of chronic stress in the last 12 months, respectively. With the LTE, participants have to indicate if a certain life event occurred. The questionnaire consists of 12 items and a higher score means that more life events occurred. The LDI uses 12 questions about life aspects, where participants have to indicate how stressful these life aspects were. The score ranges from 0-24, and a higher score indicates more stress in the past 12 months.

**Table S1. Physical activity and sedentary time of participants with and without Raynaud’s phenomenon**

|  | Raynaud’s phenomenon (N=856) | No Raynaud’s phenomenon (N=17570) |  |
| --- | --- | --- | --- |
|  | **Median (IQR)** | **Median (IQR)** | **p** |
| Total physical activity (min/week) | 2580 (1555 – 3345) | 2550 (1580 – 3340) | .643 |
| Low physical activity (min/week) | 1980 (1040 – 2848) | 2070 (1110 – 2880) | .118 |
| Moderate physical activity (min/week) | 180 (60 – 450) | 180 (60 – 420) | .204 |
| Vigorous physical activity (min/week) | 45 (0 – 140) | 0 (0 – 120) | **<.001** |
| MVPA (min/week) | 300 (140 – 610) | 255 (120 – 540) | **<.001** |
| Commuting (min/week) | 15 (0 – 100) | 5 (0 – 75) | **<.001** |
| Work (min/week) | 900 (0 – 1680) | 840 (0 – 1620) | .240 |
| Household (min/week) | 840 (420 – 1380) | 900 (470 – 1530) | **<.001** |
| Leisure time (min/week) | 390 (220 – 630) | 360 (210 – 600) | .057 |
| Guidelines 150 min MVPA/week (%yes) | 636 (74%) | 12213 (70%) | **.003** |
| Sitting weekday (min/day) | 540 (360 – 720)† | 540 (360 – 690)‡ | .914 |

MVPA = moderate to vigorous physical activity, IQR=Interquartile Range †n=367, ‡n=6897

1 Wendel-Vos GC, Schuit AJ, Saris WH, Kromhout D (2003) Reproducibility and relative validity of the short questionnaire to assess health-enhancing physical activity. J Clin Epidemiol 56(12):1163-1169. <https://doi.org/10.1016/s0895-4356(03)00220-8>

2 Ainsworth BE, Haskell WL, Herrmann SD, Meckes N, Bassett DR, Jr., Tudor-Locke C, Greer JL, Vezina J, Whitt-Glover MC, Leon AS (2011) 2011 Compendium of Physical Activities: a second update of codes and MET values. Med Sci Sports Exerc 43(8):1575-1581. <https://doi.org/10.1249/MSS.0b013e31821ece12>

3 (2020) WHO Guidelines Approved by the Guidelines Review Committee. WHO Guidelines on Physical Activity and Sedentary Behaviour, World Health Organization

© World Health Organization 2020., Geneva

4 Marshall AL, Miller YD, Burton NW, Brown WJ (2010) Measuring total and domain-specific sitting: a study of reliability and validity. Med Sci Sports Exerc 42(6):1094-1102. <https://doi.org/10.1249/MSS.0b013e3181c5ec18>

5 Aaronson NK, Muller M, Cohen PD, Essink-Bot ML, Fekkes M, Sanderman R, Sprangers MA, te Velde A, Verrips E (1998) Translation, validation, and norming of the Dutch language version of the SF-36 Health Survey in community and chronic disease populations. J Clin Epidemiol 51(11):1055-1068. <https://doi.org/10.1016/s0895-4356(98)00097-3>

6 Brugha TS, Cragg D (1990) The List of Threatening Experiences: the reliability and validity of a brief life events questionnaire. Acta Psychiatr Scand 82(1):77-81. <https://doi.org/10.1111/j.1600-0447.1990.tb01360.x>

7 Hendriks A, Ormel J, van de Willige G (1990) Long-term difficulties measured by a self-report questionnaire and semi-structured interview: a comparison of methods. Gedrag en Gezondheid 18:273-283
